# Synthesis and application of gas diffusion cathodes in an advanced type of undivided electrochemical cell

**DOI:** 10.1038/s41598-020-74199-2

**Published:** 2020-10-14

**Authors:** S. Z. J. Zaidi, Y. Luan, C. Harito, L. Utari, B. Yuliarto, F. C. Walsh

**Affiliations:** 1 Electrochemical Engineering Laboratory, Energy Technology Research Group, Faculty of Engineering and Environment, Engineering Sciences, University of Southampton, Highfield, Southampton, SO17 1BJ UK; 2 Institute of Chemical Engineering and Technology, University of the Punjab, Lahore, Pakistan; 3 Industrial Engineering Department, Faculty of Engineering, Bina Nusantara University, Jakarta, 11480 Indonesia; 4 Advanced Functional Materials (AFM) Laboratory, Engineering Physics, Institut Teknologi Bandung, 40132 Bandung, Indonesia; 5 Research Center for Nanosciences and Nanotechnology (RCNN), Institut Teknologi Bandung, 40132 Bandung, Indonesia

**Keywords:** Environmental sciences, Nanoscale materials

## Abstract

This paper reports the oxidation of Remazol black B dye by employing iron ions catalyst based gas diffusion cathodes, (GDCs). A GDC was synthesized by using a layer of carbon black and iron ions catalyst for oxygen reduction to hydrogen peroxide. The results demonstrated around 97% decolorization of Remazol black-B dye for 50 min by iron ions catalyst based GDC. The degradation study was performed under electrogenerated hydrogen peroxide at a constant voltage of − 0.6 V *vs* Hg/HgSO_4_ in which the rate of degradation was correlated with hydrogen peroxide production. Overall, the GDC’s found to be effective method to degrade the dyes via electro-Fenton.

## Introduction

The waste water containing organic dyes which cause toxic effects on human health and damage aquatic natural resources. A large amount of organic dyes are being used in food industry^1–3^, leather tanneries^4–6^, pharmaceuticals^7,8^ and in textile industry^9–13^.

The oxidation and removal of dyes from wastewater have been investigated by methods like chemical^14^, photoelectrochemical^15^, adsorption^16–20^, membrane technology^21–23^ and advanced oxidation processes^24^. The electrochemical generation of hydrogen peroxide by oxygen reduction reaction (ORR) for the electrochemical water treatment with subsequent formation of ^·^OH radical by using electro Fenton has been utilized for water purification and mineralisation for over last three decades^25^. Currently, Photo Fenton^26^, Electro Fenton^27,28^, Photo Electro Fenton^29,30^ have been employed for the electrochemical synthesis of hydrogen peroxide. It is evident from the literature that it is much needed to develop an inexpensive electrode that may provide better efficiency and employability in terms of performance and reusability. Moderate cost electrodes like RVC have been employed for the electrophoretic deposition of titanate nanosheets (TiNS) for anodic water treatment^31^. Likewise, low cost electrodes can also be synthesized by using carbon materials for electro Fenton treatment of wastewater containing dyes. Gas diffusion electrodes (GDC) can be an effective alternative electrode for the electroFenton synthesis. GDC modified with nitrogen doped graphene at carbon nanotube electrodes have been reported as effective for the generation of 25 mg dm^−3^ of H_2_O_2_ at an applied potential of − 0.5 V *vs.* standard calomel electrode (SCE)^32^. The study to improve GDC modified with Fe (II) catalyst showed about 99% decolorization of methylene blue in 250 min at − 1.0 V *vs.* Hg/HgSO_4_^33^. Nitrogen functionalised carbon nanotube cathodes was observed to be useful for complete degradation of methyl orange at − 0.85 V in 60 min^29^. In this work, hydrogen peroxide production and mineralisation have been studied with the improved GDC electrodes and evaluate the oxidation of Remazol black–B dye in an advanced type of electrochemical cell.

## Experimental

Carbon based cloth, namely EC-AC-Cloth-T, was procured from QuinTech Technology, Berlin, Germany. Reagent grade nickel mesh, 0.05 mm thickness (a current collector) acquired from Dexmet Corporation, Wallingford, USA and Carbon black VULCAN^®^ XC72R obtained from Cabot, Boston, USA. Polytetrafuoroethylene (Teflon^®^ PTFE *DISP 30*) was obtained from *DuPont*™, UK. Reagent grade Na_2_SO_4_, acetone, and KMnO_4_ were received from Fischer Scientific (Loughborough, UK) while Remazol Black-B and ethanol was obtained from Sigma Aldrich (Gillingham, UK) and employed as received. All aqueous solutions were made by using double distilled water acquired from a distillation equipment provided by SUEZ Water UK (Peterborough, UK), exhibiting resistivity less than 17 MΩ cm at 25 °C.

### Synthesis of gas diffusion electrodes

**Figure 1:**
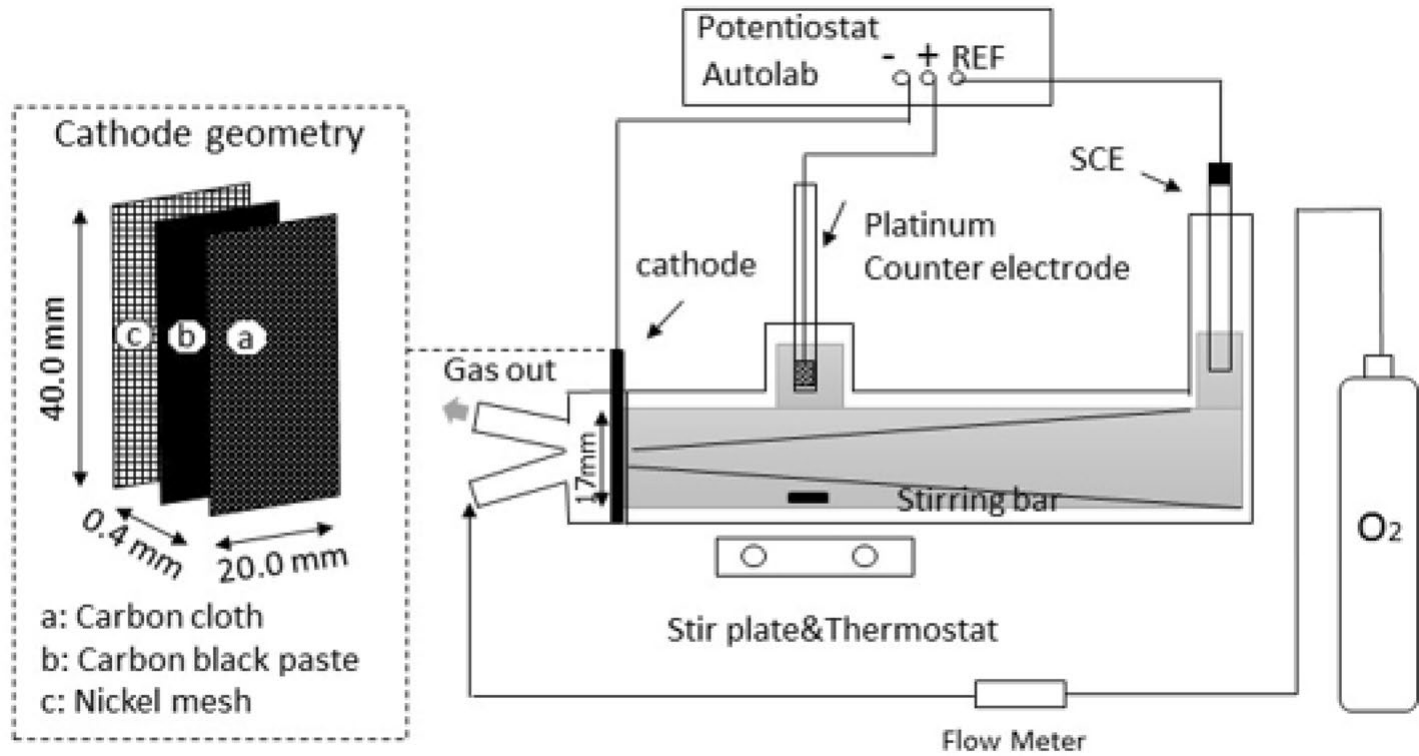
GDC electrodes geometry and experimental arrangement of the undivided electrochemical flow cell.

The improved gas diffusion cathodes (GDCs) were made by adopting method previously reported in the literature^34^. Similarly, GDC comprised of catalytic carbon and Ni mesh for current collector. A paste for catalyst layer was produced from 25 mg carbon black and 125 mg FeCl_2_. Ultrasonic bath (XUBA3, Grant Instruments, UK) was used to sonicate the mixture of carbon black and FeCl_2_ for 10 min in 5 wt% Nafion^®^ from Alfa Aesar, UK (D-520 dispersion). The resulting sonicated paste of Nafion, carbon black, and FeCl_2_ was rolled over 40 mm × 40 mm × 0.4 mm on a piece of carbon cloth pre-treated with 25 wt% PTFE as shown in Fig. 1. The catalyst loading was found around 5 mg cm^−2^ on the electrode.

Finally, electrode hot-pressed by using Carver 3851-0 Hydraulic Press (Cole Palmer, UK) for 2 min at 140 °C under a pressure of 5 MPa by arranging a 0.02 mm thick nickel mesh (current collector) as a top cover for the layered carbon cloth with carbon black and FeCl_2_ layer. The GDC electrodes were recovered from the hydraulic press and allowed to spontaneously cool down in air for 10 min.

### Characterization of GDC

The characterization of the as prepared GDC electrodes was conducted by employing JEOL JSM-6500F field emission scanning electron microscope (FESEM) at an accelerating voltage of 10–15 kV. The surface morphology of the carbon black and iron ions catalyst layer carbon cloth and nickel mesh layer were observed by using FESEM.

### Electrochemical study

The electrochemical experiments were carried out as our previous studies^34^ in an undivided advanced type of three electrode electrochemical cell (Fig. 1) by using high-end potentiostat/galvanostat PGSTAT302 N (Metrohm Autolab B.V., Utrecht, Netherlands) and analysed by Nova 1.11. A 0.80 cm^2^ working electrode made from iron ions modified GDC was deployed. Meanwhile, Pt mesh was employed as counter electrode with SCE (i.e., Hg/HgSO_4_) as reference electrode. A 0.15 L solution containing 17 ppm of Remazol black-B dye was made in 0.5 mol L^3^ of Na_2_SO_4_ solution as background electrolyte. The electrolysis was conducted at − 0.6 V and − 0.7 V *vs.* Hg/HgSO_4_ in an undivided electrochemical batch reactor with oxygen supply (flow rate = 0.1 dm^3^ min^−1^) across the gas diffusion electrodes.

A UV–Vis spectrophotometer Hitachi U3010 (Abingdon, UK) was used to measure the absorbance at 597 nm as the peak of Remazol black-B dye. Beer–Lambert law was applied for calibration and calculation of the Remazol black-B concentration. Further, demineralization studies of the Remazol black-B dye were performed using Shimadzu TOC–V_CPH_ TOC detector. Using the standard nonpurgeable organic carbon (NPOC) analysis, TOC values were measured.

## Results and discussion

### Surface characterization of GDC electrodes

**Figure 2:**
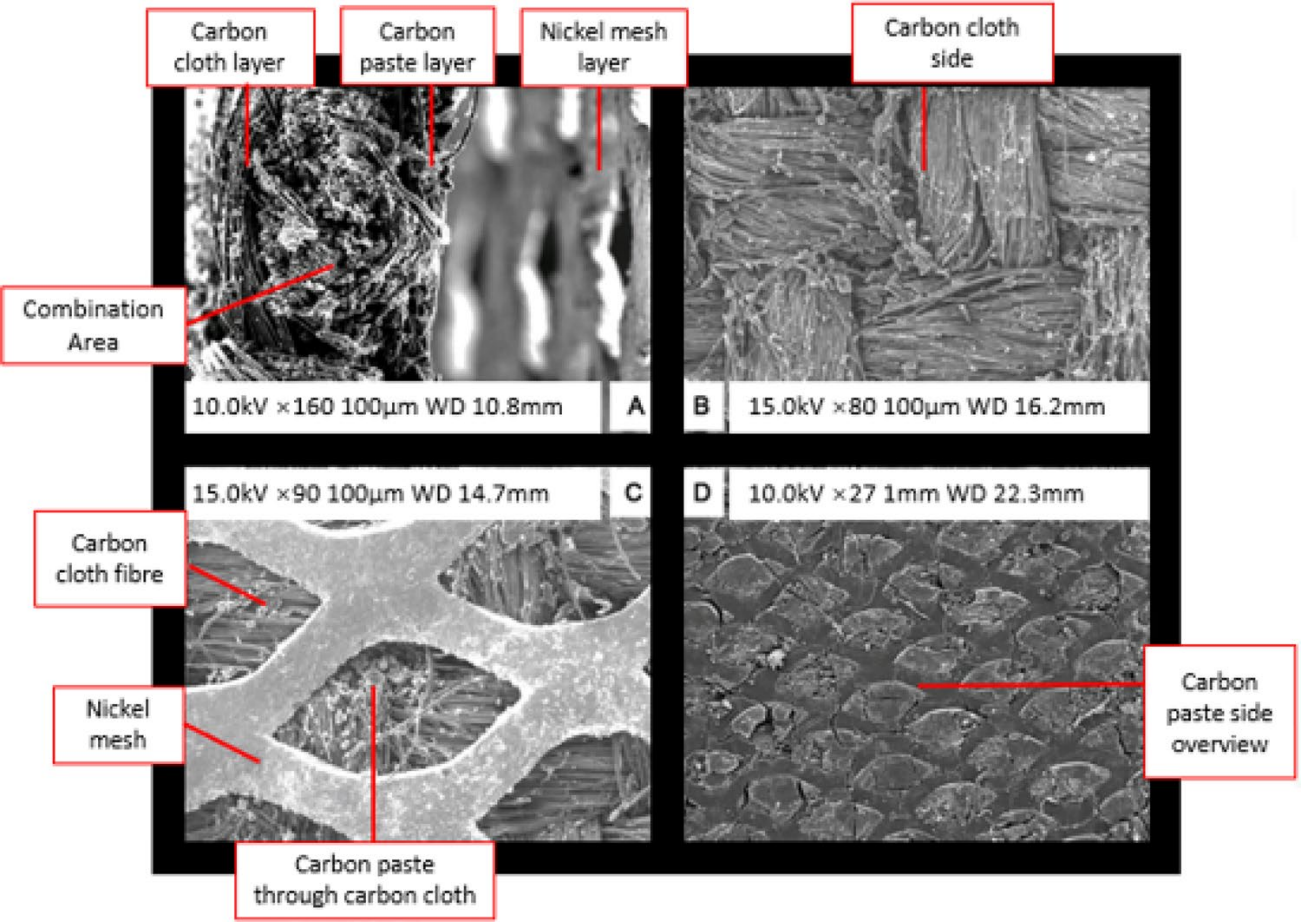
SEM pictures of GDC electrodes (**A**) Over all combination area of GDC electrodes. (**B**) Carbon cloth side view. (**C**) Nickel mesh top view. (**D**) Carbon paste and iron ions side view.

Figure 2A–D exhibits the representative images of the gas diffusion electrodes taken by using FESEM. Figure 2A shows the carbon and catalyst layer, nickel mesh and carbon cloth. Figure 2B depicts the side view of the carbon cloth fibers. Figure 2C shows the top view of the GDC electrodes across the nickel mesh which shows the carbon layer and catalyst layer over the surface of carbon cloth. Figure 2D represents the top view of the carbon and catalyst paste over the carbon cloth. It can be seen that the layer of carbon black and iron ions catalyst were successfully adhered over the carbon cloth surface. The FeCl_2_ catalyst was transformed into a mixture of FeOCl and Fe_2_O_3_ in aqueous solution following the Eqs. (1) and (2)^35^.

1 $$4{\text{FeCl}}_{2} + { }2{\text{H}}_{2} {\text{O}} + {\text{ O}}_{2} \to { }4{\text{FeOCl }} + { }4{\text{HCl}}$$

2 $$4{\text{FeCl}}_{2} + { }4{\text{H}}_{2} {\text{O}} + {\text{ O}}_{2} \to { }2{\text{Fe}}_{2} {\text{O}}_{3} { } + { }8{\text{HCl}}$$

While FeCl_2_ is soluble in aqueous solution, FeOCl and Fe_2_O_3_ have limited solubility in water^36^. Hence these improved iron ions catalyst based GDC electrodes may provide better performance in comparison to other reported electrodes.

### Polarization curve studies for the ORR

The GDC electrodes were mounted in an advanced type of electrochemical batch cell (Fig. 1) to conduct the polarization studies for oxygen reduction reaction (ORR). The experiments were conducted in a 0.5 mol dm^−3^ Na_2_SO_4_ at pH 3 by feeding pure oxygen at 0.1 dm^3^ min^−1^ across gas diffusion electrodes at scan rate of 10 mVs^−1^ in range of − 0.1 to − 1.0 V *vs.* Hg/HgSO_4_. The dissolved oxygen supplied across the GDC electrodes was electrochemically transformed into hydrogen peroxide via the (ORR) as shown in Eq. (3). However, it has been reported in the literature studies related to ORR that in this process, two side competing reactions also occurs as a secondary reaction^37^. These two side reactions are shown in Eq. (4) i.e. reduction of hydrogen peroxide to water and hydrogen gas evolution as depicted in Eq. (5):

3 $${\text{O}}_{2} + 2{\text{H}}^{ + } + { }2{\text{e}}^{ - } \to {\text{H}}_{2} {\text{O}}_{2} { }E^\circ = 0.695{\text{ V }}vs.{\text{ SHE }}$$

4 $${\text{H}}_{2} {\text{O}}_{2} + 2{\text{H}}^{ + } + { }2{\text{e}}^{ - } \to 2{\text{H}}_{2} {\text{O }}E^\circ { } = { }1.763{\text{ V }}vs.{\text{ SHE}}$$

5 $$2{\text{H}}^{ + } { } + { }2{\text{e}}^{ - } \to {\text{ H}}_{{2\left( {\text{g}} \right)}} E^\circ { } = { }0.00{\text{ V }}vs.{\text{ SHE}}$$

**Figure 3:**
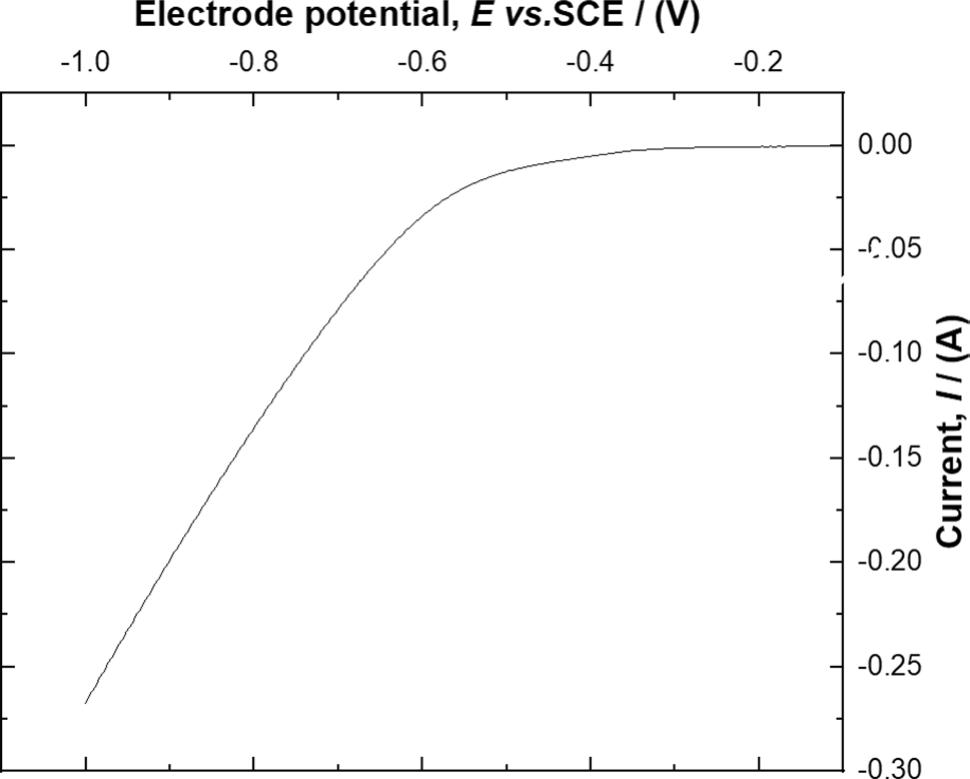
Polarisation curve at GDC electrodes with iron ion for the determination of ORR in a solution containing in 0.5 mol dm^–3^ Na_2_SO_4_ potential sweep rate = 10 mV s^−1^; temperature: 25 °C, oxygen flowrate 0.1 dm^3^ min^−1^.

Figure 3 showed when the potential was sweep at a scan rate of 10 mVs^−1^, a steep rise in cathodic current was observed after − 0.5 V *vs.* Hg/HgSO_4_. The cathodic polarization curve indicates that the ORR takes place in potential range of − 0.3 to − 0.6 V *vs.* Hg/HgSO_4_, this corresponding region for limiting current indicates the optimal cathodic region for the oxygen reduction to hydrogen peroxide. When the potential was sweep and continues to rise above − 0.6 V *vs.* Hg/HgSO_4_, the current increases steadily which representing the region of the competing side reactions as indicated by Eqs. (4) and (5).

### Hydrogen peroxide concentration accumulation during electrolysis

**Figures 4:**
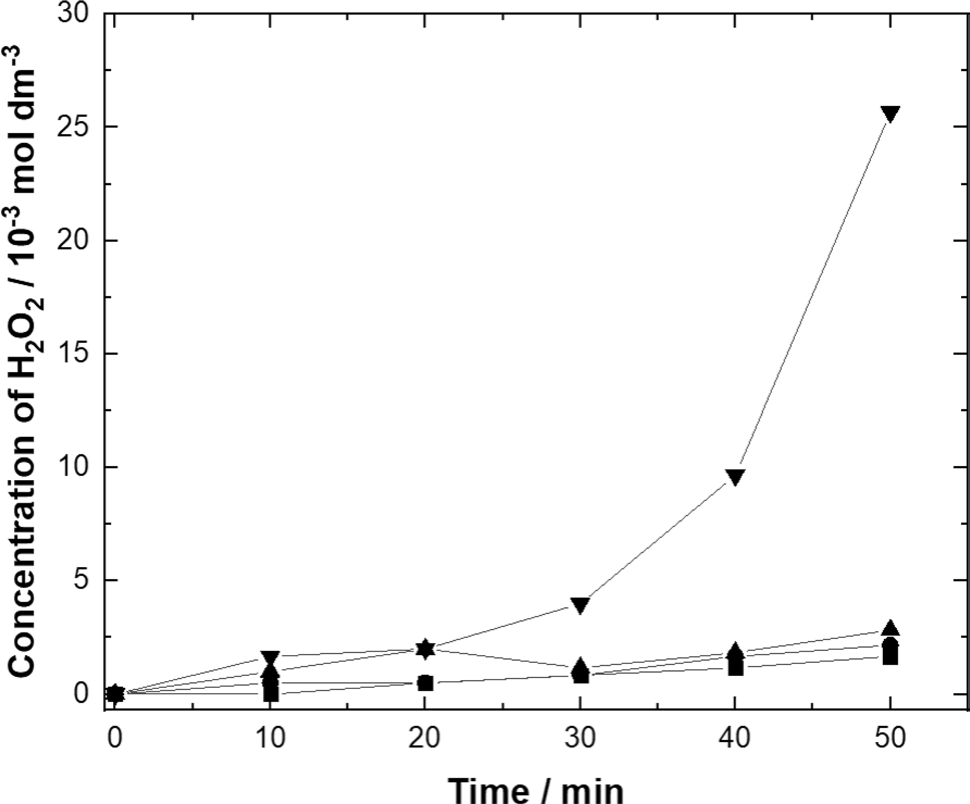
Accumulation of electrogenerated hydrogen peroxide during the electrolysis at different electrode potentials on GDC electrodes (filled square) − 0.3 V *vs.* Hg/HgSO_4_, (filled circle) − 0.4 V *vs.* Hg/HgSO_4_, (filled triangle) − 0.5 V *vs.* Hg/HgSO_4_, (filled inverted triangle) − 0.6 V *vs.* Hg/HgSO_4_ in an electrolyte containing in 0.5 mol dm^–3^ Na_2_SO_4_ pH   3 temperature: 25 °C, oxygen flowrate 0.1 dm^3^ min^−1^.

The generation of hydrogen peroxide during ORR is the most important step during the electroFenton process. The constant potential studies were conducted to measure the accumulation of hydrogen peroxide. The studies were carried out in the potential range of − 0.3 V to − 0.6 V *vs.* Hg/HgSO_4_ in a 0.5 mol dm^−3^ Na_2_SO_4_ solution at pH 3. The results were shown in Fig. 4. It can be seen that the concentration of hydrogen peroxide was increased as the cathode potential rise from − 0.3 to − 0.6 V *vs.* Hg/HgSO_4_. The results seem to be in agreement with the polarization curve as shown in Fig. 3. So, for the decolorization studies of the Remazol black-B dye the electrolysis at − 0.6 V *vs.* Hg/HgSO_4_ seems to be desirable for electro Fenton process.

### Decolorization of Remazol black-B by using iron ions based GDC electrodes

After the successfully generating hydrogen peroxide at − 0.6 V *vs.* Hg/HgSO_4_, the decolorization of Remazol black-B dye was performed by applying constant potential electrolysis at − 0.6 V *vs.* Hg/HgSO_4_. The oxidation of Reactive Black 5 dye was assisted by powerful oxidizing agent hydroxyl radicals created from the breakup of electrogenerated H_2_O_2_ in the presence of Fe^3+^ and propagated by Fe^2+^ ions over the surface of GDC electrodes. The generation of hydroxyl radical mechanism at the Fe^2+^ and Fe^3+^ based GDC electrodes is depicted in Eqs. (6) and (7)^38^;

6 $${\text{Fe}}^{3 + } + {\text{ H}}_{2} {\text{O}}_{2} \to {\text{Fe}}^{2 + } + {\text{H}}^{ + } + {\text{HO}}_{2}^{ \cdot }$$

7 $${\text{Fe}}^{2 + } + {\text{ H}}_{2} {\text{O}}_{2} \to {\text{Fe}}^{3 + } + {\text{OH}}^{ - } + ^{ \cdot } {\text{OH }}$$

A 150 cm^3^ of electrolyte containing 17 mg dm^−3^ of Remazol black-B dye and 0.5 mol dm^−3^ of Na_2_SO_4_ at pH  3 was used under constant potential electrolysis at − 0.6 V *vs.* Hg/HgSO_4_ with working electrode (GDC), counter electrode (Pt), and reference electrode (Hg/HgSO_4_). Samples were collected at a regular interval of 10 min for constant potential electrolysis studies.

**Figure 5:**
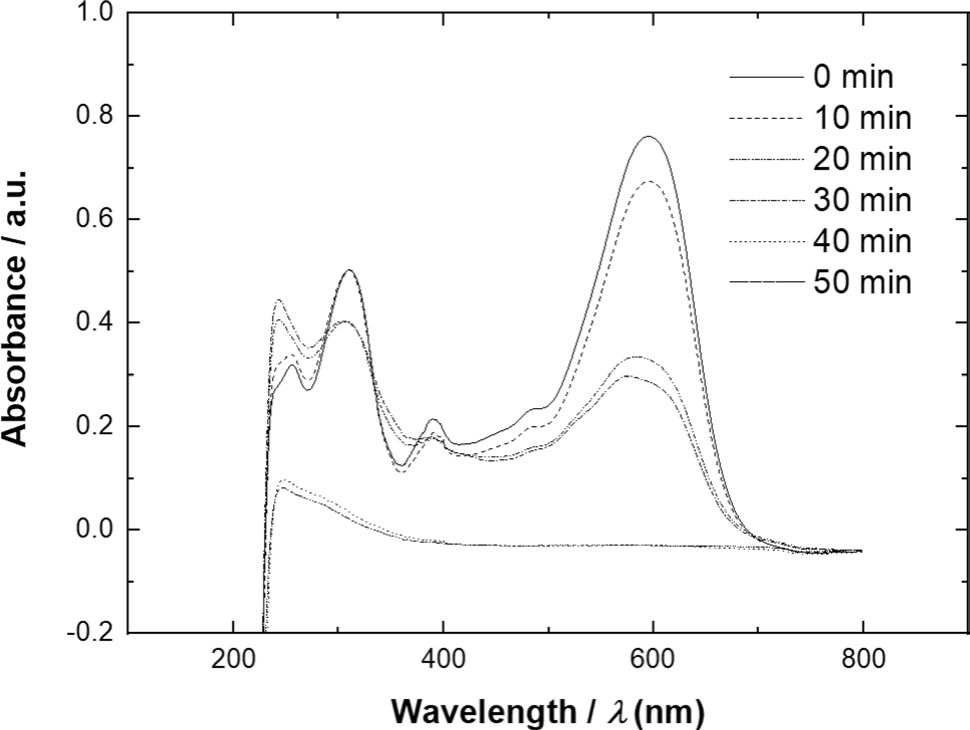
UV–Vis spectra of samples collected during 50 min electrolysis on an iron ions based GDC electrodes by applying constant potential of − 0.7 V *vs.* Hg/HgSO_4_ from a solution containing 17 mg dm^−3^ Remazol black B dye 0.5 mol dm^−3^ Na_2_SO_4_ as a background electrolyte at 25 °C, oxygen flowrate 0.1 dm^3^ min^−1^, pH 3.

Figure 5 shows the spectrum obtained from UV spectrophotometer which displayed the absorbance *vs.* wavelength of the samples withdrawn during electrolysis studies for the decolorization of Remazol black-B dye at a constant potential of − 0.7 V *vs.* Hg/HgSO_4_. Initially, the concentration of dye was 17 mg dm^−3^. The dye spectra at this point was represented by continuous line having 2 absorption peaks, which is the visible spectrum region at 597 nm and the UV spectrum region at 311 nm. The absorbance at a wavelength of 597 nm is related with the azo group (N=N) of Remazol black-B while at a wavelength of 311 nm is related with the aromatic groups^34^. It can be seen in that after 40 min of electrolysis was sufficient to eliminate the peaks related to azo group and aromatic groups in the spectrum of absorbance *vs.* wavelength.

**Figure 6:**
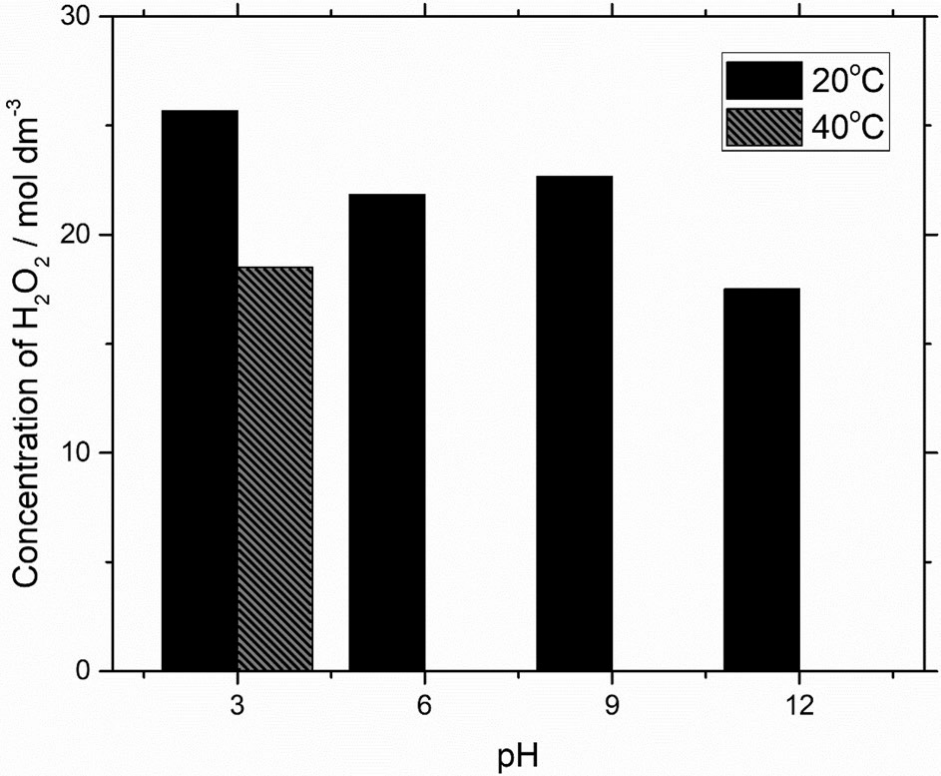
Effect of pH on hydrogen peroxide during the electrolysis at different electrode potentials on an iron ions GDC electrodes by applying constant potential of − 0.7 V *vs.* Hg/HgSO_4_ in an electrolyte containing in 0.5 mol dm^–3^ Na_2_SO_4_.

In Fig. 6, the effect of temperature and pH on the hydrogen peroxide production was recorded at − 0.7 V *vs.* Hg/HgSO_4_ to find the optimum condition. It was found that the production of hydrogen peroxide was optimum at 20 °C in pH 3 solution. High temperature, 40 °C, and high pH (pH > 5) may degrade the hydrogen peroxide hence less concentration was detected^39,40^. The hydrogen peroxide decomposed into oxygen and hydrogen in which the reaction was highly influenced by temperature, pH, and the use of catalyst. It was also reported that most of the commercial product was kept below pH 5 to preserve the hydrogen peroxide^41^.

**Figure 7:**
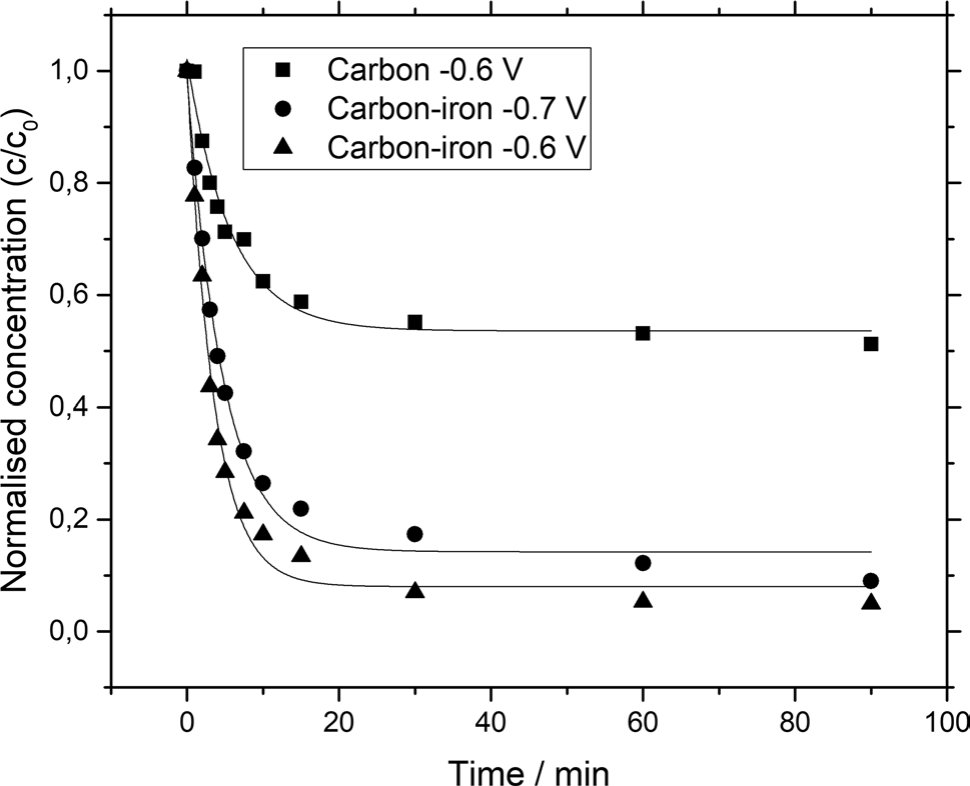
Electro Fenton oxidation of Remazol black B dye using (filled square) carbon only at − 0.6 V *vs.* Hg/HgSO_4_, and using iron ions based GDC at (filled circle) − 0.6 V *vs.* Hg/HgSO_4_, (filled triangle) − 0.7 V *vs.* Hg/HgSO_4_.

Figure 7 displayed the impact of the electrode potential on the decolorization of Remazol black B during the constant potential electrolysis of electrolyte containing 17 mg dm^−3^ of Remazol black B and 0.5 mol dm^−3^ Na_2_SO_4_ at pH   3. The results showed that the decolorization studies at two potentials − 0.6 and − 0.7 V *vs.* Hg/HgSO_4_. The impact of the applied potential on the degradation of Remazol black B is due to the oxygen reduction of hydrogen peroxide species in which the iron ions assist the well-known electro Fenton reaction^34,37^ by promoting powerful ^·^OH radical as represented in Eq. (4).

The Fig. 7 depicted that the dye oxidation seems to be 97% after 50 min at the electrode potential of − 0.6 V *vs.* Hg/HgSO_4_. This behaviour is in the agreement with polarization curves results presented in Fig. 3 which shows the region of highest hydrogen peroxide at − 0.6 V *vs.* Hg/HgSO_4_. When the decolorization studies were carried out at − 0.7 V *vs.* Hg/HgSO_4_, it was observed that the decolorization decreased which obviously means the evolution of side reactions which competes with the oxygen reduction reaction. Since, the electrode was coated with the immobilized iron ions which initiate the classical electro Fenton reaction producing the ^·^OH radical which attacks the Remazol black-B to breakdown into intermediates. The comparison of the rate constant at different electrode potential displayed that using − 0.6 V *vs.* Hg/HgSO_4_ oxidized 97% of the Remazol black-B dye. Some studies related to photo electro Fenton studies exhibited 70% conversion of RB-5 dye^42^ which seems to be quite lower in comparison to these studies as represented in Table1. Notice that the colour removal of the dye decreased by increasing the applied voltage to − 0.7 V *vs.* Hg/HgSO_4_ which caused by the competing secondary reactions occurred over the electrode surface.

**Table 1:** Comparison of decolorization of Remazol black B dye by other related values from selected literature.

Substrate	% Decolouration	Time/min	References
GDC containing iron ions as catalyst	97	50	This study
PbO_2_/RVC	97	60	^30^
Calcined TiO_2_/PbO_2_/RVC	98	60	^30^
Photoassisted Fenton by using iron oxide on activated alumina support	70	480	^42^
Photoassisted Fenton by using GO/NiFe_2_O_4_	98	30	^43^
Isolated bacterial *Pseudomonas aeruginosa*	100	1920	^44^
NAR-2 modified HDTMA-Br	95.87	40	^45^

### Demineralization of Remazol black-B by using iron ions based GDC electrodes

**Figure 8:**
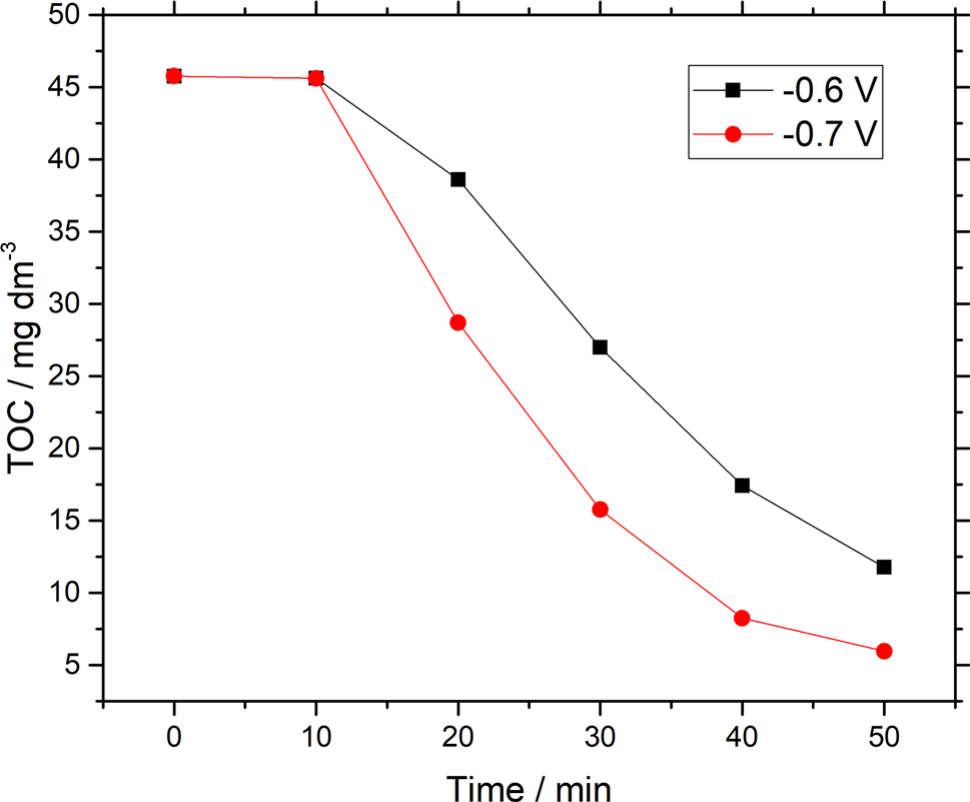
Effect of different potentials on demineralization of Remazol black-B dye via TOC measurements *vs.* electrolysis time by using iron ions based GDC at (filled square) − 0.6 V *vs.* Hg/HgSO_4_, (filled circle) − 0.7 V *vs.* Hg/HgSO_4_.

The TOC abatement studies of Remazol black B dye was conducted to account for the demineralization of dye. For this study, constant potentials at − 0.6 and − 0.7 V vs. Hg/HgSO_4_ were opted for electro Fenton demineralization of Remazol black B dye. The results are presented in the Fig. 8, it can be observed that degradation of Remazol black-B dye was initiated by the decomposition of electrogenerated H_2_O_2_ which in turn produce ^·^OH radical formed in the presence of Fe^2+^ and Fe^3+^ ions at the top of Fe-based GDC cathodes. The demineralization of Remazol black-B dye was shown and drop of TOC from 45  to 5 mg dm^−3^ by performing electrolysis at fixed potential of − 0.7 V vs. Hg/HgSO_4_ as seen in Fig. 8. Demineralization was decreased while applying potentials at − 0.6 V vs. Hg/HgSO_4_.

This represented that the application of greater constant potential (− 0.7 V vs. Hg/HgSO_4_) demineralized about 88% of the total organic carbon content present in the solution of Remazol black-B dye as shown in Fig. 8.

**Figure 9:**
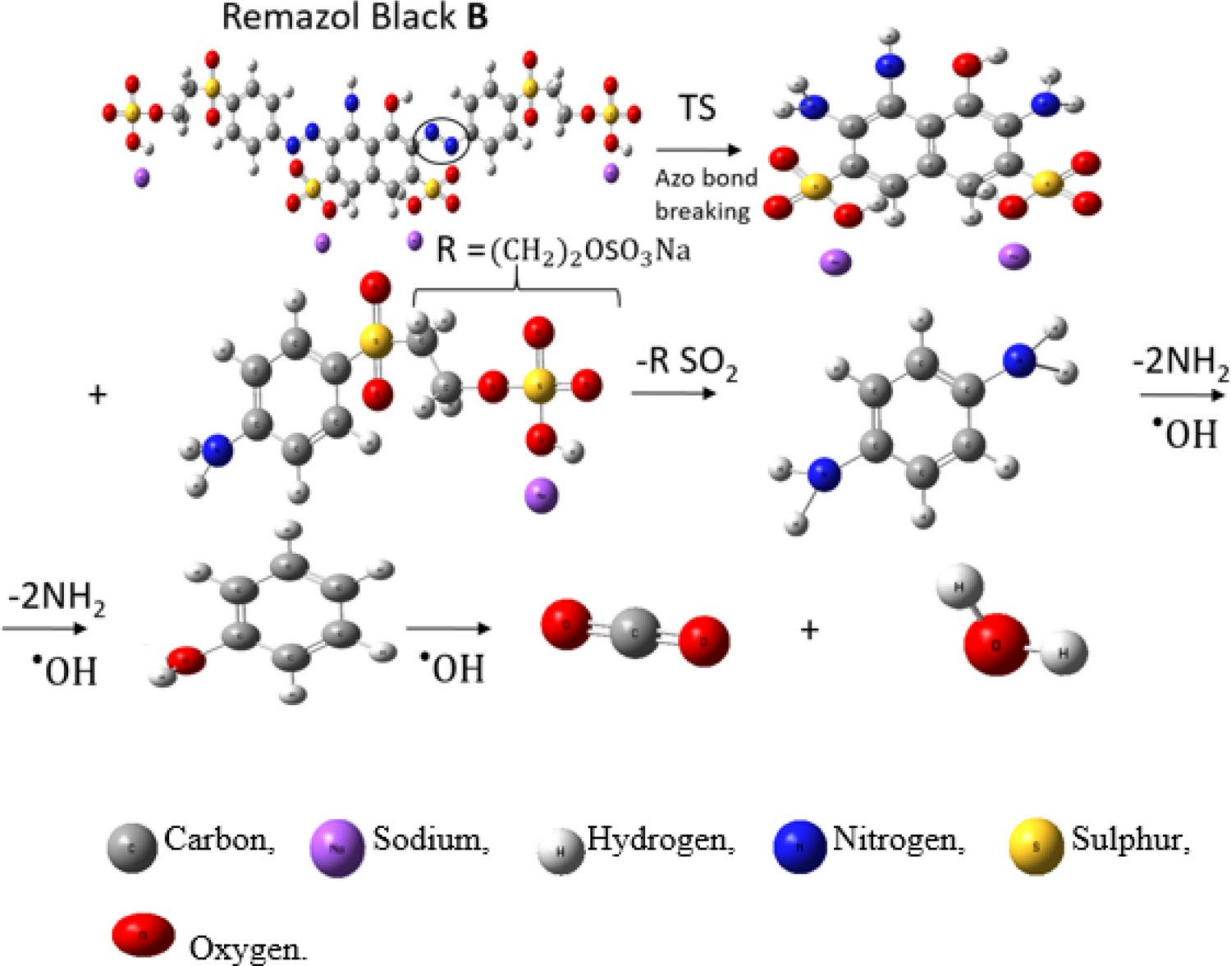
Degradation mechanism pathway for the oxidation of Remazol black B dye.

The proposed route for the degradation pathway of Remazol black-B is shown in Fig. 9. The proposed route was associated with the breaking of the aromatic heterocyclic chain (azo bond) of Remazol black-B producing transitory intermediates. The intermediates were further converted into benzene amino sulphonic acid in the presence of the ^·^OH radical by removing R = (CH_2_)_2_OSO_3_Na^38^. The addition of the ^·^OH radical to amino benzene sulphonic acid resulted in the generation of phenol molecule by removing the NH_2_ group. Finally, Remazol black-B dye was transformed into aliphatic acidic compounds, water and carbon dioxide.

### Current efficiency and energy consumption for the demineralization of Remazol black-B dye using iron ions based GDC electrodes

The ratio of electric energy employed for electrochemical decomposition reaction to total current passed through the electrochemical process is known as current efficiency.

The following equation is used to measure the current efficiency (CE) for an electrochemical demineralization reaction^34^:

8 $${\mathbf{CE}} = \frac{{2.67\left[ {{\mathbf{TOC}}_{0} - {\mathbf{TOC}}_{{\mathbf{f}}} } \right]\user2{ FV}}}{{8\user2{ }\mathop \smallint \nolimits_{{}}^{t} Idt\user2{ t}}} \times 100$$

where 2.67 is the reported equivalent factor of COD (Chemical Oxygen Demand) to TOC, (TOC_0_) and (TOC_f_) related to the TOC (g dm^−3^) values at the initial and final time of electrolysis respectively, F is the Faraday constant (96,500 C mol^−1^), V is the volume of the working solution (dm^3^), 8 is the factor for oxygen equivalent mass (g eq^−1^), $\mathop \smallint \limits_{0}^{t} Idt$ overall current passed in an electrochemical demineralization and t is the overall time interval (s) for the electrolysis reaction.

**Figure 10:**
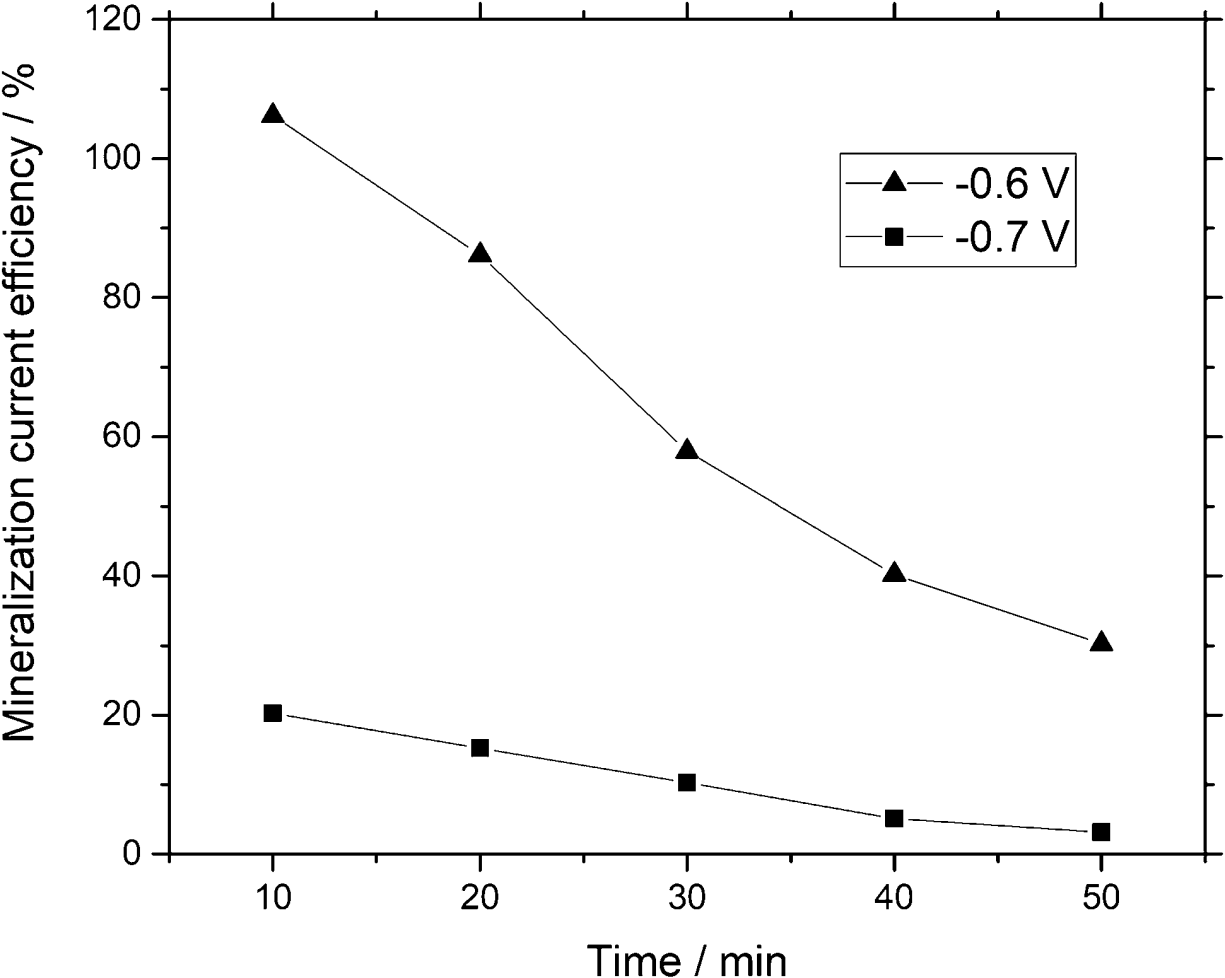
Current efficiency related to mineralization of Remazol black-B dye calculated from Eq. (9) *vs.* electrolysis time by using iron ions based GDC at (filled triangle) − 0.6 V *vs.* Hg/HgSO_4_, (filled square) − 0.7 V *vs.* Hg/HgSO_4_.

The current efficiency is influenced by TOC abatement analysis, time of electrochemical reaction and total current utilized. Figure 10 shows the current efficiency *versus* electrolysis time. At − 0.6 V vs. Hg/HgSO_4_, the current efficiency obtained after 30 min of electrolysis reaction was about 60% and gradually decreased with time to 34% as calculated by using Eq. (8). At − 0.7 V vs. Hg/HgSO_4_, the highest current efficiency after 30 min of constant potential electrolysis was about 17% and gradually decrease to 3% at the end of electrolysis. The decreasing trends in CE during the electrolysis was due to the difficulty to degrade more aromatic compounds into inorganic compounds, which ultimately produce CO_2_. However, the appearance of side reactions like hydrogen evolution may lead to less current efficiency at higher applied potential.

The energy consumption, EC, (kWh kg^−1^TOC) for the electrolysis process was calculated by the equation given below^34^;

9 $${\text{EC}} = { }\frac{{E I_{o} \Delta t}}{{100 ({\text{TOC}}_{0} - {\text{TOC}}_{{\text{f}}} ) V}}$$

where *E* (V) is the potential at which electrolysis reaction takes place, *I*_*o*_ (A) is the current detected during electrolysis reaction. Meanwhile, time of electrolysis is denoted as ∆*t* (h). (TOC_0_) and (TOC_f_) are the TOC (kg dm^−3^) values at start and at the end of electrochemical reaction respectively and *V* (dm^3^) relates with the volume of the electrolyte containing electrochemical species. Likewise, EC increased as forecasted, as it required greater electric energy in an electrolysis process to convert intermediate organic products as a break down compounds of Remazol black -B dye.

**Figure 11:**
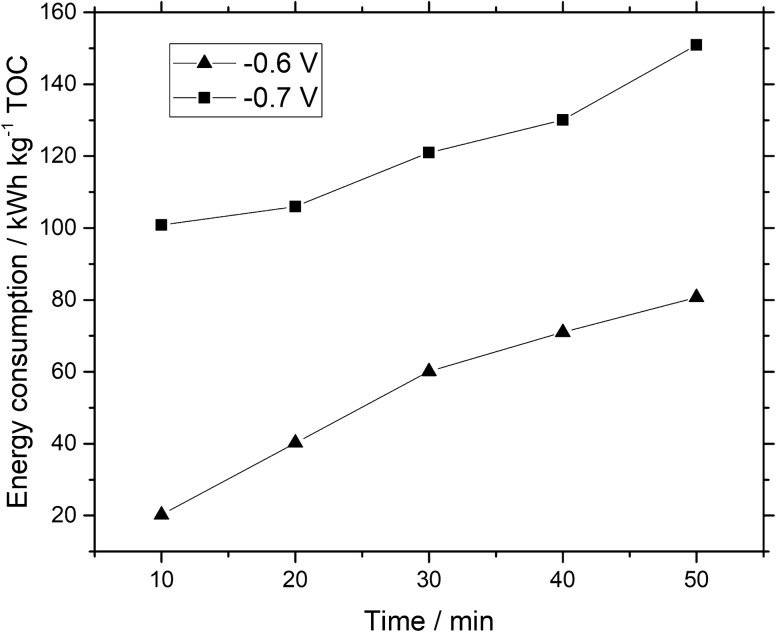
Energy consumption related to mineralization of Remazol black-B dye calculated from Eq. (9) *vs.* electrolysis time by using iron ions based GDC at (filled triangle) − 0.6 V *vs.* Hg/HgSO_4_, (filled square) − 0.7 V *vs.* Hg/HgSO_4_.

After 50 min, the energy consumption calculated using Eq. (9) was about 157 kWh kg^−1^ TOC at − 0.7 V *vs.* Hg/HgSO_4_ as shown in Fig. 11. Note that the energy consumption for decomposition of Remazol black B dye decreased as applied constant potential decreased to − 0.6 V vs. Hg/HgSO_4_.

## Conclusions

In this work an improved GDC electrodes has been produced by rolling a layer of C black with iron ion species for the oxidation of Remazol black B dye. The polarization studies showed − 0.6 V *vs.* Hg/HgSO_4_ founds to be feasible for the hydrogen peroxide production. It was demonstrated that 25 mmol dm^−3^ hydrogen peroxide was produced by ORR in an advanced type of undivided cell by applying an electrode potential of − 0.6 V *vs.* Hg/HgSO_4_. GDC containing iron ions can effectively oxidize the Remazol black-B dye with 99% decolorization and demineralization of 88%. The current efficiency (CE) was found to be 17% after 30 min of electrolysis at − 0.7 V *vs.* Hg/HgSO_4_. The energy consumption (EC) was 157 kWh kg^−1^ TOC by applying constant potential difference of − 0.7 V *vs.* Hg/HgSO_4._ The results suggested the significance of immobilized iron ions over GDC electrodes for the removal of toxic Remazol black-B dye in an advanced type of 3 electrode electrochemical batch cell.

## References

[1] Roy K, Gullapalli S, Roy Chaudhuri U, Chakraborty R (2004). The use of a natural colorant based on betalain in the manufacture of sweet products in India. Int. J. Food Sci. Technol..

[2] Barros WRP, Steter JR, Lanza MRV, Tavares AC (2016). Catalytic activity of Fe3-xCuxO4 (0≤x≤0.25) nanoparticles for the degradation of Amaranth food dye by heterogeneous electro-Fenton process. Appl. Catal. B Environ..

[3] Hernández-Martínez AR (2017). Stability comparison between microencapsulated red-glycosidic pigments and commercial FD&C Red 40 dye for food coloring. J. Mater. Sci..

[4] Masoud RA, Haroun AA, El-Sayed NH (2006). Dyeing of chrome tanned collagen modified by in situ grafting with 2-EHA and MAC. J. Appl. Polym. Sci..

[5] Ramalingam S, Sreeram KJ, Raghava Rao J, Unni Nair B (2016). Organic nanocolorants: Self-fixed, optothermal resistive, silica-supported dyes for sustainable dyeing of leather. ACS Sustain. Chem. Eng..

[6] Ramalingam S, Jonnalagadda RR (2017). Tailoring nanostructured dyes for auxiliary free sustainable leather dyeing application. ACS Sustain. Chem. Eng..

[7] Wainwright M (2008). Dyes in the development of drugs and pharmaceuticals. Dyes Pigm..

[8] Pérez-Ibarbia L, Majdanski T, Schubert S, Windhab N, Schubert US (2016). Safety and regulatory review of dyes commonly used as excipients in pharmaceutical and nutraceutical applications. Eur. J. Pharm. Sci..

[9] Türgay O, Ersöz G, Atalay S, Forss J, Welander U (2011). The treatment of azo dyes found in textile industry wastewater by anaerobic biological method and chemical oxidation. Sep. Purif. Technol..

[10] Hayat H, Mahmood Q, Pervez A, Bhatti ZA, Baig SA (2015). Comparative decolorization of dyes in textile wastewater using biological and chemical treatment. Sep. Purif. Technol..

[11] Raman CD, Kanmani S (2016). Textile dye degradation using nano zero valent iron: A review. J. Environ. Manage..

[12] Paździor K (2017). Influence of ozonation and biodegradation on toxicity of industrial textile wastewater. J. Environ. Manage..

[13] Dias FF, Oliveira AAS, Arcanjo AP, Moura FCC, Pacheco JGA (2016). Residue-based iron catalyst for the degradation of textile dye via heterogeneous photo-Fenton. Appl. Catal. B Environ..

[14] Larouk S (2017). Catalytic ozonation of Orange-G through highly interactive contributions of hematite and SBA-16—To better understand azo-dye oxidation in nature. Chemosphere.

[15] Meshram N (2019). Effect of tetravalent ions dopants and CoOx surface modification on hematite nanorod array for photoelectrochemical degradation of Orange-II dye. J. Taiwan Inst. Chem. Eng..

[16] Tang WW (2012). Simultaneous adsorption of atrazine and Cu (II) from wastewater by magnetic multi-walled carbon nanotube. Chem. Eng. J..

[17] Hao J, Ji L, Li C, Hu C, Wu K (2018). Rapid, efficient and economic removal of organic dyes and heavy metals from wastewater by zinc-induced in-situ reduction and precipitation of graphene oxide. J. Taiwan Inst. Chem. Eng..

[18] Tsai MJ, Wu JY (2019). Insight into the influence of framework metal ion of analogous metal–organic frameworks on the adsorptive removal performances of dyes from water. J. Taiwan Inst. Chem. Eng..

[19] Stan M (2019). Starch-coated green synthesized magnetite nanoparticles for removal of textile dye Optilan Blue from aqueous media. J. Taiwan Inst. Chem. Eng..

[20] Gorza FDS (2018). Electrospun polystyrene-(emeraldine base) mats as high-performance materials for dye removal from aqueous media. J. Taiwan Inst. Chem. Eng..

[21] Yu L, Han M, He F (2017). A review of treating oily wastewater. Arab. J. Chem..

[22] Hou C (2017). Preparation of TiO2 nanoparticles modified electrospun nanocomposite membranes toward efficient dye degradation for wastewater treatment. J. Taiwan Inst. Chem. Eng..

[23] Hebbar RS, Isloor AM, Zulhairun AK, Sohaimi Abdullah M, Ismail AF (2017). Efficient treatment of hazardous reactive dye effluents through antifouling polyetherimide hollow fiber membrane embedded with functionalized halloysite nanotubes. J. Taiwan Inst. Chem. Eng..

[24] Martínez-Huitle CA, Brillas E (2009). Decontamination of wastewaters containing synthetic organic dyes by electrochemical methods: A general review. Appl. Catal. B.

[25] Lacasa E, Cañizares P, Walsh FC, Rodrigo MA, Ponce-de-León C (2019). Removal of methylene blue from aqueous solutions using an Fe2+ catalyst and in-situ H2O2 generated at gas diffusion cathodes. Electrochim. Acta.

[26] Li Y, Chen D, Fan S, Yang T (2019). Enhanced visible light assisted Fenton-like degradation of dye via metal-doped zinc ferrite nanosphere prepared from metal-rich industrial wastewater. J. Taiwan Inst. Chem. Eng..

[27] Ren G, Zhou M, Liu M, Ma L, Yang H (2016). A novel vertical-flow electro-Fenton reactor for organic wastewater treatment. Chem. Eng. J..

[28] Es’haghzade Z, Pajootan E, Bahrami H, Arami M (2017). Facile synthesis of Fe3O4 nanoparticles via aqueous based electro chemical route for heterogeneous electro-Fenton removal of azo dyes. J. Taiwan Inst. Chem. Eng..

[29] Salazar R, Garcia-Segura S, Ureta-Zañartu MS, Brillas E (2011). Degradation of disperse azo dyes from waters by solar photoelectro-Fenton. Electrochim. Acta.

[30] Salazar R, Ureta-Zañartu MS (2012). Mineralization of triadimefon fungicide in water by electro-Fenton and photo electro-Fenton. Water. Air Soil Pollut..

[31] Zaidi SZJ, Harito C, Walsh FC, de León CP (2018). Decolourisation of reactive black-5 at an RVC substrate decorated with PbO2/TiO2 nanosheets prepared by anodic electrodeposition. J. Solid State Electrochem..

[32] Liu T (2016). New electro-Fenton gas diffusion cathode based on nitrogen-doped graphene@carbon nanotube composite materials. Electrochim. Acta.

[33] McKerracher RD (2016). A high-performance, bifunctional oxygen electrode catalysed with palladium and nickel-iron hexacyanoferrate. Electrochim. Acta.

[34] Zaidi SZJ, Hurter E, Walsh FC, de León CP (2019). Fe(II)-based GDE electrodes for the demineralization of methylene blue dye. Arab. J. Sci. Eng..

[35] Suganuma K, Yagi T (1978). Thermal decomposition of iron(II) chloride. Nippon Kagaku Kaishi.

[36] Yang XJ, Xu XM, Xu J, Han YF (2013). Iron oxychloride (FeOCl): An efficient Fenton-like catalyst for producing hydroxyl radicals in degradation of organic contaminants. J. Am. Chem. Soc..

[37] De Leon CP, Pletcher D (1995). Removal of formaldehyde from aqueous solutions via oxygen reduction using a reticulated vitreous carbon cathode cell. J. Appl. Electrochem..

[38] Popli S, Patel UD (2017). Mechanistic aspects of electro-catalytic reduction of Reactive black 5 dye in a divided cell in the presence of silver nano-particles. Sep. Purif. Technol..

[39] Crole DA, Freakley SJ, Edwards JK, Hutchings GJ (2016). Direct synthesis of hydrogen peroxide in water at ambient temperature. Proc. R. Soc. A Math. Phys. Eng. Sci..

[40] Yazici EY, Deveci H, Gülsoy ÖY, Ergün LŞ, Canfrffg NM, Çelik İB (2010). Factors affecting decomposition of hydrogen peroxide. Proceedings of the XIIth International Mineral Processing Symposium.

[41] Evonik. *Stability & Decomposition*. https://active-oxygens.evonik.com/product/h2o2/en/about-hydrogen-peroxide/general-information/stability-and-decomposition/. Accessed 9 Apr 2020.

[42] Hsueh CL, Huang YH, Wang CC, Chen CY (2006). Photoassisted Fenton degradation of nonbiodegradable azo-dye (Reactive black 5) over a novel supported iron oxide catalyst at neutral pH. J. Mol. Catal. A Chem..

[43] Sheshmani S, Falahat B, Nikmaram FR (2017). Preparation of magnetic graphene oxide-ferrite nanocomposites for oxidative decomposition of Remazol black B. Int. J. Biol. Macromol..

[44] Hashem RA, Samir R, Essam TM, Ali AE, Amin MA (2018). Optimization and enhancement of textile reactive Remazol black B decolorization and detoxification by environmentally isolated pH tolerant *Pseudomonas aeruginosa* KY284155. AMB Express.

[45] Kardi SN, Rashid NAA, Ibrahim N, Ahmad A (2016). Biodegradation of Remazol black B in sequential microaerophilic–aerobic operations by NAR-2 bacterial consortium. Environ. Earth Sci..

